# Crystal structure of diethyl 2-amino-5-{4-[bis­(4-methyl­phen­yl)amino]­benzamido}­thio­phene-3,4-di­carboxyl­ate

**DOI:** 10.1107/S2056989019003864

**Published:** 2019-04-09

**Authors:** Yohan Gautier, Thierry Maris, W. G. Skene

**Affiliations:** a Département de chimie, Université de Montréal, CP 6128, Centre-ville Montreal, QC, H3C 3J7, Canada

**Keywords:** crystal structure, intra- and inter­molecular bonding, hydrogen-bonded dimer

## Abstract

In the title compound, C_31_H_31_N_3_O_5_S, the regioselective substitution of the thio­phene is confirmed with the amine and the amide at the 2- and 5-positions, respectively. In the mol­ecule, the thio­phene ring is twisted by 12.82 (3)° with respect to the aromatic ring of the benzamido group. Intra­molecular N—H⋯O hydrogen bonds are present involving the N atoms of the primary amine and the amide groups, forming *S*(6) ring motifs. In the crystal, centrosymmetrically related mol­ecules are linked by pairs of N—H⋯O hydrogen bonds involving the amide carbonyl O atoms and the primary amine N atoms to form dimers of *R*_2_^2^(16) ring motif.

## Abstract 2

The title compound forms a head-to-head centrosymmetric dimer, involving a pair of inter­molecular N—H⋯O hydrogen bonds. It also forms two intra­molecular bonds between its amine and amide and the ester carbonyl groups.

## Chemical context   

Azomethines are prepared by the condensation of amines with aldehydes. Using aromatic precursors, the reaction results in the preparation of conjugated azomethines having colors that are readily detectable in the visible spectrum (Dufresne *et al.*, 2007 ▸). This is particularly the case with azomethines that are prepared from 2,5-di­amino­thio­phene derivatives (Bolduc *et al.*, 2013 ▸). These derivatives can be electrochemically oxidized (Yeh *et al.*, 2016 ▸). The collective properties (reversible color change with applied potential) have proven ideal for use as electrochromic materials (Ma *et al.*, 2016 ▸). While various azomethines have been studied for understating the impact of structure on the absorption and electrochemical properties (Liu *et al.*, 2018 ▸), modifying the terminal amine has remained relatively underexplored. Such modification allows property tuning, including reversible oxidation. This is a key property for electrochromic use. Given the underexplored modification of 2-amino­thio­phenes, we investigated its conversion to a tri­phenyl­amide. The tri­phenyl­amide moiety was targeted because of its electrochemically reversible oxidation. Meanwhile, the amide functional group was chosen because of its robustness that could sustain electrochemical redox cycles. More importantly, it would be inert towards imination reactions for constructing conjugated azomethines having both various terminal groups and cores. Given the challenge of unequivocally identifying the configuration and absolute structural identification of amino­thio­phene derivatives with the concomitant limited number of reported tri­phenyl­amine amides, the X-ray crystal structure analysis of the title compound (**I**) was evaluated and it is reported on herein.
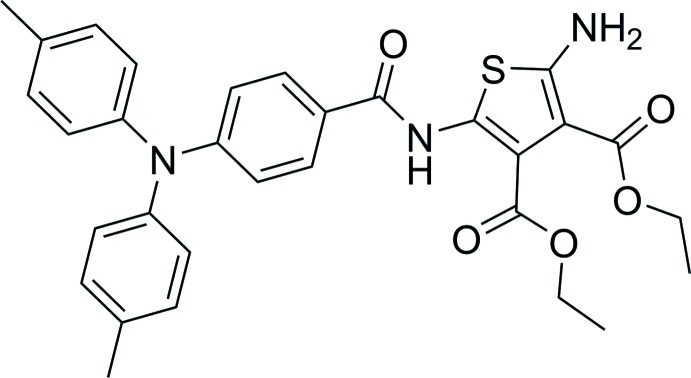


## Structural commentary   

**Figure 1:**
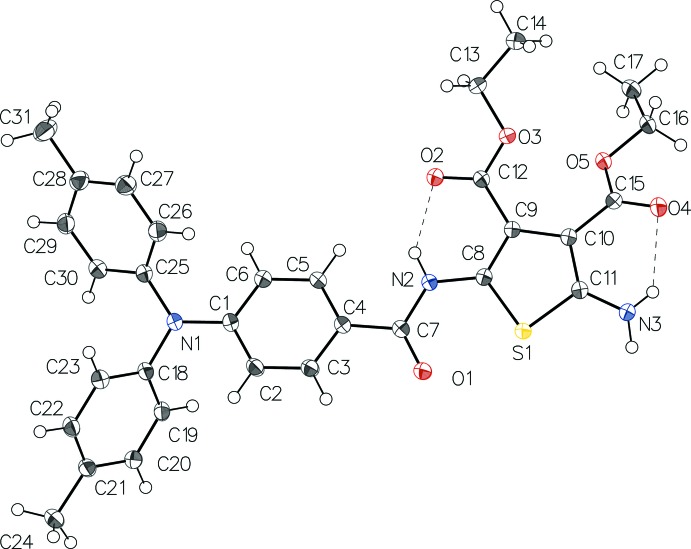
The mol­ecular structure of **I** with displacement ellipsoids drawn at the 50% probability level. H atoms are shown as spheres of arbitrary radius. Intra­molecular hydrogen bonds are shown as dashed lines.

In the mol­ecule of **I** (Fig. 1 ▸), the mean plane through the 2,5-di­amino­tihophene ring (r.m.s. deviation = 0.0116 Å) is inclined to the C1–C6 benzene (ring *A*) by 12.82 (3)°. The dihedral angles formed by the benzene rings *A*, *B* (C18–C23) and *C* (C25–C30) of the tri­phenyl­amide moiety are: *A*^*B* = 65.56 (3)°, *A*^*C* = 55.22 (4)°, *B*^*C* = 66.80 (4)°. The O1—C7, N2—C7, N2—C8 and N3—C11 bond lengths are 1.2315 (13), 1.3644 (13), 1.3829 (13) and 1.3529 (14) Å, respectively. While the reactivity of the primary amine of **I** is less than that expected for typical aryl­amines owing to the electron-withdrawing esters, it nonetheless acts as a hydrogen donor. In fact, two N—H⋯O intra­molecular hydrogen bonds occur, one each between the ester carbonyl and its adjacent nitro­gen, forming rings of *S*(6) graph-set motif (Table 1 ▸). The intra­molecular hydrogen bonds observed are consistent with those reported in other 2-amino-3-ester thio­phenes (Dufresne & Skene, 2010*a* ▸,*b* ▸; Skene *et al.*, 2006 ▸; Bourgeaux & Skene, 2007 ▸; Bourgeaux *et al.*, 2006 ▸; Bolduc *et al.*, 2010 ▸; Tshibaka *et al.*, 2011 ▸; Furuyama *et al.*, 2014 ▸). The crystal structure of **I** confirms the asymmetric substitution of thio­phene by a primary amine and an amide. Of importance is that the thio­phene substitution with the nitro­gen atoms occurs at the 2,5-positions, rather than the 3,4-positions. The primary amine at the 2-thio­phene position is also confirmed. The 2,5-configuration is desired because extended degrees of conjugation result when the azomethines are formed in these positions with aryl­amines. The presence of ester functionalities at the 3,4-positions is also verified by the crystal structure.

## Supra­molecular features   

**Figure 2:**
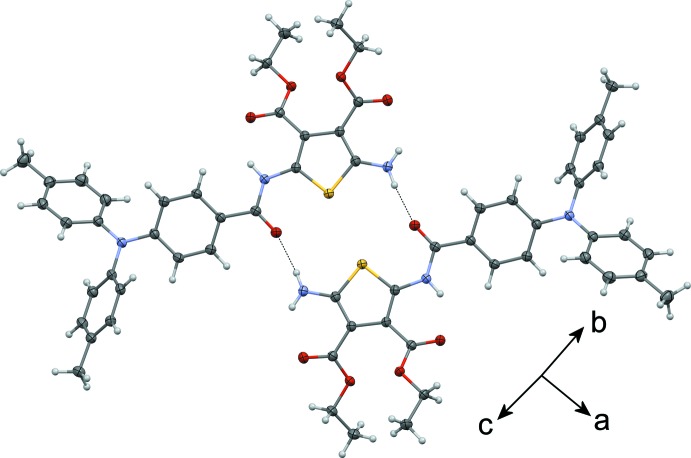
Supra­molecular dimer of **I** showing the inter­molecular hydrogen bonds as dotted lines.

**Figure 3:**
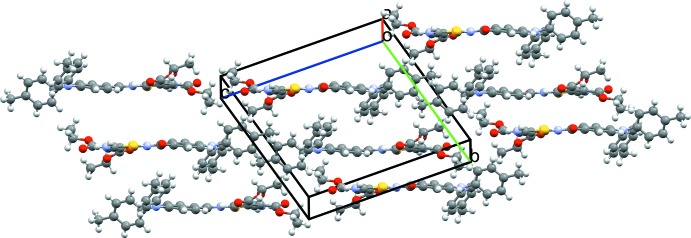
Crystal packing of **I** approximately viewed along the *a* axis.

In the crystal structure of **I**, centrosymmetrically related mol­ecules are linked into head-to-head hydrogen-bonded dimers (Fig. 2 ▸) by pairs of N—H⋯O hydrogen bonds (Table 1 ▸) involving the N3 amine atom and the O1 carbonyl atom of the amide group, forming rings of 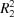
(16) graph-set motif. In this arrangement, the sulfur atoms of the two thio­phenes are face-to-face and the two heteoratoms are separated by 3.5419 (4) Å. The crystal packing (Fig. 3 ▸) is further stabilized by van der Waals forces.

## Database survey   

A survey of the Cambridge Structural Database (CSD, Version 5.39, latest update August 2018; Groom *et al.*, 2016 ▸) yielded no hits. In fact, no exact thio­phene derivatives substituted in the 3,4-positions with electron-withdrawing groups were found. Four structurally similarly thio­phenes were identified, three of which were symmetric with amides at the 2,5-positions (refcodes LOFTAD, LOFTEH, LOFTIL; Fabbro *et al.*, 2014 ▸). The most closely related structure was the asymmetric 2-amino, 5-phenyl­amido-thio­phene derivative (LOYDIM; Rodinovskaya *et al.*, 2002 ▸). No differences greater than 3σ were found for the N2—C7, N2—C8, and O1—C7 bond lengths of **I** and the nine counterpart bonds for the reported similar structures. The notable difference was the C11—N3 bond length of **I**, which is 0.025 Å (3σ = 0.004 Å) shorter than the corresponding bond in LOYDIM [1.378 (5) Å]. The dihedral angle between the planes described by the phenyl­amide and the 2,5-di­amino­tihophene rings is also different [5.74 (13)°]. The database survey yielded only four 4-amido-tri­phenyl­amines [GUWNAP, GUWNET (Ghosh *et al.*, 2009 ▸), and UZEXAZ (Wang *et al.*, 2011 ▸)], with one being complexed with cerium (ZOKSUP; Sun *et al.*, 2014 ▸). No differences between the N1—phenyl and C4—C7 bond distances were found. The three phenyl-N-phenyl dihedral angles of **I** are also consistent with the those of the reported structures.

## Synthesis and crystallization   

To a solution of 4-(di-*p*-tolyl­amino)­benzoic acid (668 mg, 1.7 mmol, 1 eq) in anhydrous di­chloro­methane (15 mL) were added oxalyl chloride (0.21 mL, 2.3 mmol, 1.8 eq) and one drop of anhydrous DMF. The mixture was stirred for 16 h under nitro­gen at room temperature. The solvent was removed under reduced pressure and the resulting 4-(di-*p*-tolyl­amino)­benzoyl chloride was dissolved in anhydrous THF (20 mL). The mixture was then added dropwise to a solution of diethyl 2,5-di­amino­thio­phene-3,4-di­carboxyl­ate (594 mg, 2.3 mmol, 1.1 eq) and Et_3_N (2.3 mmol, 0.32 mL, 1.1 eq) in anhydrous THF (5 mL). The reaction mixture was stirred for 6 h under nitro­gen at room temperature. After filtering, the solvent of the filtrate was removed under reduced pressure. The residue was purified by SiO_2_ column chromatography (hexa­nes/ethyl acetate 2:1 *v*/*v*) to afford the title compound as a yellow solid (589 mg, yield 64%). A suitable crystal of the title compound was obtained by slow evaporation of deuterated chloro­form from an NMR tube. ^1^H NMR (400 MHz, CDCl_3_): δ = 11.31 (*s*, 1H), 7.72 (*d*, ^3^ *J* = 8.9 Hz, 2H), 7.13 (*d*, ^3^ *J* = 8.2 Hz, 4H), 7.05 (*d*, ^3^ *J* = 8.2 Hz, 4H), 6.97 (*d*, ^3^ *J* = 8.9 Hz, 2H), 5.67 (*s*, 2H), 4.29 (*m*, 4H), 2.34 (*s*, 6H), 1.34 (*t*, ^3^ *J* = 7.12 Hz, 3H), 1.32 (*t*, ^3^ *J* = 7.1 Hz, 3H). ^13^C NMR (100 MHz, CDCl_3_): δ = 166.2, 165.5, 163.4, 154.6, 152.3, 143.9, 136.4, 134.6, 130.4, 128.7, 126.2, 122.5, 119.0, 109.2, 101.8, 61.0, 60.2, 21.0, 14.5, 14.3. MS–HR: (*M* + H^+^) exp . *m*/*z* = 558.2062, (*M* + H^+^) calc. *m*/*z* = 558.2057.

## Refinement   

Crystal data, data collection and structure refinement details are summarized in Table 2 ▸. The amine H atoms were located in a difference-Fourier map and refined freely. All other H atoms were placed geometrically and refined with C—H = 0.95–0.99 Å, and with *U*_iso_(H) = 1.2*U*_eq_(C) or 1.5*U*_eq_(C) for methyl H atoms. A rotating model was used for the methyl groups.

## Supplementary Material

Crystal structure: contains datablock(s) I. DOI: 10.1107/S2056989019003864/rz5251sup1.cif

Structure factors: contains datablock(s) I. DOI: 10.1107/S2056989019003864/rz5251Isup2.hkl

Supporting information file. DOI: 10.1107/S2056989019003864/rz5251Isup3.cml

CCDC reference: 1904525

Additional supporting information:  crystallographic information; 3D view; checkCIF report

## Figures and Tables

**Table 1:** Hydrogen-bond geometry (Å, °)

*D*—H⋯*A*	*D*—H	H⋯*A*	*D*⋯*A*	*D*—H⋯*A*
N2—H2⋯O2	0.850 (17)	1.958 (17)	2.6501 (12)	137.8 (15)
N3—H3*A*⋯O1^i^	0.883 (17)	2.154 (17)	3.0316 (13)	172.5 (14)
N3—H3*B*⋯O4	0.810 (17)	2.156 (16)	2.7656 (13)	132.2 (14)

Symmetry code: (i) 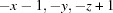.

**Table 2:** Experimental details

Crystal data	
Chemical formula	C_31_H_31_N_3_O_5_S
*M*_r_	557.65
Crystal system, space group	Triclinic, *P* 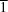
Temperature (K)	100
*a*, *b*, *c* (Å)	7.1314 (2), 13.4650 (3), 15.4586 (4)
α, β, γ (°)	106.533 (1), 97.980 (1), 102.843 (1)
*V* (Å^3^)	1354.61 (6)
*Z*	2
Radiation type	Ga *K*α, λ = 1.34139 Å
μ (mm^−1^)	0.94
Crystal size (mm)	0.16 × 0.11 × 0.04

Data collection	
Diffractometer	Bruker Venture Metaljet
Absorption correction	Multi-scan (*SADABS*; Krause *et al.*, 2015 ▸)
*T*_min_, *T*_max_	0.679, 0.752
No. of measured, independent and observed [*I* > 2σ(*I*)] reflections	43601, 6212, 5916
*R*_int_	0.024
(sin θ/λ)_max_ (Å^−1^)	0.650

Refinement	
*R*[*F* ^2^ > 2σ(*F* ^2^)], *wR*(*F* ^2^), *S*	0.036, 0.097, 1.05
No. of reflections	6212
No. of parameters	377
H-atom treatment	H atoms treated by a mixture of independent and constrained refinement
Δρ_max_, Δρ_min_ (e Å^−3^)	0.39, −0.19

Computer programs: *APEX2* and *SAINT* (Bruker, 2013 ▸), *SHELXT* (Sheldrick, 2015*a* ▸), *SHELXL2018* (Sheldrick, 2015*b* ▸), *OLEX2* (Dolomanov *et al.*, 2009 ▸), *Mercury* (Macrae *et al.*, 2008 ▸), *PLATON* (Spek, 2009 ▸), and *publCIF* (Westrip, 2010 ▸).

## supplementary crystallographic information

### Crystal data

**Table:**

C_31_H_31_N_3_O_5_S	*Z* = 2
*M**_r_* = 557.65	*F*(000) = 588
Triclinic, *P*1	*D*_x_ = 1.367 Mg m^−^^3^
*a* = 7.1314 (2) Å	Ga *K*α radiation, λ = 1.34139 Å
*b* = 13.4650 (3) Å	Cell parameters from 9687 reflections
*c* = 15.4586 (4) Å	θ = 2.7–60.6°
α = 106.533 (1)°	µ = 0.94 mm^−^^1^
β = 97.980 (1)°	*T* = 100 K
γ = 102.843 (1)°	Block, yellow
*V* = 1354.61 (6) Å^3^	0.16 × 0.11 × 0.04 mm

### Data collection

**Table:**

Bruker Venture Metaljet diffractometer	6212 independent reflections
Radiation source: Metal Jet, Gallium Liquid Metal Jet Source	5916 reflections with *I* > 2σ(*I*)
Helios MX Mirror Optics monochromator	*R*_int_ = 0.024
Detector resolution: 10.24 pixels mm^-1^	θ_max_ = 60.7°, θ_min_ = 2.7°
ω and φ scans	*h* = −9→9
Absorption correction: multi-scan (SADABS; Krause *et al.*, 2015)	*k* = −17→17
*T*_min_ = 0.679, *T*_max_ = 0.752	*l* = −20→20
43601 measured reflections	

### Refinement

**Table:**

Refinement on *F*^2^	Primary atom site location: dual
Least-squares matrix: full	Hydrogen site location: mixed
*R*[*F*^2^ > 2σ(*F*^2^)] = 0.036	H atoms treated by a mixture of independent and constrained refinement
*wR*(*F*^2^) = 0.097	*w* = 1/[σ^2^(*F*_o_^2^) + (0.0563*P*)^2^ + 0.4554*P*] where *P* = (*F*_o_^2^ + 2*F*_c_^2^)/3
*S* = 1.05	(Δ/σ)_max_ = 0.001
6212 reflections	Δρ_max_ = 0.39 e Å^−^^3^
377 parameters	Δρ_min_ = −0.18 e Å^−^^3^
0 restraints	

### Special details

**Table:**

Experimental. X-ray crystallographic data for I were collected from a single crystal sample, which was mounted on a loop fiber. Data were collected using a Bruker Venture diffractometer equipped with a Photon 100 CMOS Detector, a Helios MX optics and a Kappa goniometer. The crystal-to-detector distance was 4.0 cm, and the data collection was carried out in 1024 x 1024 pixel mode.
Geometry. All esds (except the esd in the dihedral angle between two l.s. planes) are estimated using the full covariance matrix. The cell esds are taken into account individually in the estimation of esds in distances, angles and torsion angles; correlations between esds in cell parameters are only used when they are defined by crystal symmetry. An approximate (isotropic) treatment of cell esds is used for estimating esds involving l.s. planes.

### Fractional atomic coordinates and isotropic or equivalent isotropic displacement parameters (Å^2^)

**Table:**

	*x*	*y*	*z*	*U*_iso_*/*U*_eq_	
S1	−0.27244 (4)	0.06598 (2)	0.58032 (2)	0.02122 (8)	
O1	−0.20024 (12)	0.13560 (7)	0.43721 (6)	0.02656 (18)	
O2	0.39650 (11)	0.18395 (7)	0.66336 (6)	0.02647 (18)	
O3	0.35284 (11)	0.18307 (6)	0.80411 (5)	0.02208 (16)	
O4	−0.14268 (12)	−0.01088 (7)	0.84823 (6)	0.02709 (18)	
O5	0.15438 (11)	−0.00953 (7)	0.81408 (5)	0.02333 (17)	
N1	0.44925 (14)	0.35825 (8)	0.25046 (6)	0.0242 (2)	
N2	0.07397 (14)	0.15202 (8)	0.53922 (6)	0.02104 (19)	
H2	0.199 (3)	0.1717 (13)	0.5566 (11)	0.036 (4)*	
N3	−0.42204 (14)	−0.00968 (8)	0.70555 (7)	0.0234 (2)	
H3A	−0.532 (2)	−0.0409 (13)	0.6625 (11)	0.032 (4)*	
H3B	−0.405 (2)	−0.0292 (13)	0.7503 (12)	0.031 (4)*	
C1	0.33806 (16)	0.30899 (9)	0.30286 (7)	0.0214 (2)	
C2	0.13314 (16)	0.29229 (9)	0.28679 (7)	0.0226 (2)	
H2A	0.069747	0.314057	0.239990	0.027*	
C3	0.02269 (16)	0.24461 (9)	0.33822 (7)	0.0224 (2)	
H3	−0.115902	0.233884	0.326097	0.027*	
C4	0.11076 (16)	0.21182 (9)	0.40778 (7)	0.0203 (2)	
C5	0.31471 (16)	0.22772 (9)	0.42355 (7)	0.0218 (2)	
H5	0.377465	0.205919	0.470486	0.026*	
C6	0.42706 (16)	0.27486 (9)	0.37173 (8)	0.0229 (2)	
H6	0.565238	0.284067	0.383002	0.028*	
C7	−0.01919 (16)	0.16301 (9)	0.46062 (7)	0.0209 (2)	
C8	−0.01785 (15)	0.10919 (9)	0.59882 (7)	0.0200 (2)	
C9	0.07750 (15)	0.10201 (9)	0.67966 (7)	0.0196 (2)	
C10	−0.06016 (15)	0.05446 (9)	0.72744 (7)	0.0200 (2)	
C11	−0.25434 (16)	0.03304 (9)	0.68189 (7)	0.0206 (2)	
C12	0.28951 (16)	0.15762 (9)	0.71298 (7)	0.0204 (2)	
C13	0.56112 (15)	0.23930 (9)	0.83922 (8)	0.0232 (2)	
H13A	0.587955	0.312667	0.834394	0.028*	
H13B	0.641392	0.199732	0.802979	0.028*	
C14	0.61165 (18)	0.24542 (12)	0.93859 (9)	0.0330 (3)	
H14A	0.585366	0.172379	0.942452	0.049*	
H14B	0.531150	0.284569	0.973706	0.049*	
H14C	0.751398	0.283289	0.964429	0.049*	
C15	−0.02184 (16)	0.01050 (9)	0.80248 (7)	0.0214 (2)	
C16	0.19659 (17)	−0.05354 (10)	0.88794 (8)	0.0274 (2)	
H16A	0.086332	−0.116277	0.881920	0.033*	
H16B	0.214461	0.001603	0.948819	0.033*	
C17	0.38311 (18)	−0.08751 (11)	0.87989 (9)	0.0300 (3)	
H17A	0.365227	−0.140105	0.818579	0.045*	
H17B	0.412994	−0.120184	0.927395	0.045*	
H17C	0.492230	−0.024286	0.888468	0.045*	
C18	0.35607 (16)	0.35600 (9)	0.16150 (7)	0.0220 (2)	
C19	0.24563 (16)	0.25904 (9)	0.09516 (8)	0.0226 (2)	
H19	0.231993	0.193559	0.108656	0.027*	
C20	0.15469 (16)	0.25789 (9)	0.00862 (8)	0.0236 (2)	
H20	0.074974	0.191712	−0.035401	0.028*	
C21	0.17916 (16)	0.35251 (10)	−0.01418 (8)	0.0240 (2)	
C22	0.29234 (17)	0.44882 (10)	0.05301 (8)	0.0256 (2)	
H22	0.310723	0.514013	0.038810	0.031*	
C23	0.37864 (17)	0.45147 (9)	0.14014 (8)	0.0248 (2)	
H23	0.452993	0.518152	0.185205	0.030*	
C24	0.09038 (18)	0.35211 (11)	−0.10897 (8)	0.0297 (3)	
H24A	0.195498	0.380647	−0.137308	0.045*	
H24B	−0.000735	0.397250	−0.103306	0.045*	
H24C	0.018819	0.278181	−0.147755	0.045*	
C25	0.64705 (16)	0.42384 (9)	0.28755 (8)	0.0226 (2)	
C26	0.71207 (18)	0.48747 (9)	0.38035 (8)	0.0261 (2)	
H26	0.624280	0.486611	0.421240	0.031*	
C27	0.90540 (19)	0.55223 (10)	0.41312 (9)	0.0288 (2)	
H27	0.949225	0.593205	0.476904	0.035*	
C28	1.03591 (17)	0.55826 (9)	0.35443 (9)	0.0278 (2)	
C29	0.96756 (17)	0.49614 (10)	0.26161 (9)	0.0270 (2)	
H29	1.053046	0.500286	0.220010	0.032*	
C30	0.77774 (17)	0.42840 (9)	0.22849 (8)	0.0247 (2)	
H30	0.736321	0.384840	0.165285	0.030*	
C31	1.24615 (19)	0.62789 (12)	0.39042 (11)	0.0387 (3)	
H31A	1.332337	0.583463	0.401862	0.058*	
H31B	1.255322	0.684753	0.448195	0.058*	
H31C	1.287141	0.660616	0.344560	0.058*	

### Atomic displacement parameters (Å^2^)

**Table:**

	*U*^11^	*U*^22^	*U*^33^	*U*^12^	*U*^13^	*U*^23^
S1	0.01620 (13)	0.02771 (14)	0.02086 (13)	0.00373 (10)	0.00225 (9)	0.01223 (10)
O1	0.0190 (4)	0.0387 (5)	0.0239 (4)	0.0051 (3)	0.0031 (3)	0.0160 (3)
O2	0.0181 (4)	0.0384 (5)	0.0246 (4)	0.0030 (3)	0.0041 (3)	0.0168 (3)
O3	0.0168 (4)	0.0276 (4)	0.0197 (4)	0.0006 (3)	0.0013 (3)	0.0100 (3)
O4	0.0216 (4)	0.0368 (5)	0.0266 (4)	0.0049 (3)	0.0062 (3)	0.0181 (4)
O5	0.0199 (4)	0.0296 (4)	0.0241 (4)	0.0045 (3)	0.0033 (3)	0.0166 (3)
N1	0.0211 (4)	0.0305 (5)	0.0209 (4)	0.0009 (4)	0.0023 (4)	0.0141 (4)
N2	0.0169 (4)	0.0280 (5)	0.0196 (4)	0.0034 (4)	0.0031 (3)	0.0123 (4)
N3	0.0175 (4)	0.0292 (5)	0.0241 (5)	0.0018 (4)	0.0031 (4)	0.0140 (4)
C1	0.0228 (5)	0.0229 (5)	0.0198 (5)	0.0042 (4)	0.0051 (4)	0.0101 (4)
C2	0.0234 (5)	0.0277 (5)	0.0204 (5)	0.0088 (4)	0.0043 (4)	0.0123 (4)
C3	0.0203 (5)	0.0284 (5)	0.0203 (5)	0.0076 (4)	0.0039 (4)	0.0105 (4)
C4	0.0207 (5)	0.0230 (5)	0.0175 (5)	0.0044 (4)	0.0037 (4)	0.0084 (4)
C5	0.0207 (5)	0.0257 (5)	0.0196 (5)	0.0042 (4)	0.0018 (4)	0.0112 (4)
C6	0.0193 (5)	0.0277 (5)	0.0228 (5)	0.0042 (4)	0.0032 (4)	0.0120 (4)
C7	0.0207 (5)	0.0240 (5)	0.0188 (5)	0.0055 (4)	0.0036 (4)	0.0089 (4)
C8	0.0177 (5)	0.0224 (5)	0.0209 (5)	0.0038 (4)	0.0041 (4)	0.0095 (4)
C9	0.0174 (5)	0.0221 (5)	0.0197 (5)	0.0033 (4)	0.0031 (4)	0.0095 (4)
C10	0.0180 (5)	0.0220 (5)	0.0202 (5)	0.0030 (4)	0.0036 (4)	0.0093 (4)
C11	0.0201 (5)	0.0216 (5)	0.0207 (5)	0.0042 (4)	0.0041 (4)	0.0092 (4)
C12	0.0192 (5)	0.0226 (5)	0.0211 (5)	0.0047 (4)	0.0031 (4)	0.0109 (4)
C13	0.0167 (5)	0.0259 (5)	0.0235 (5)	−0.0004 (4)	0.0006 (4)	0.0094 (4)
C14	0.0231 (6)	0.0460 (7)	0.0269 (6)	−0.0005 (5)	−0.0011 (5)	0.0182 (5)
C15	0.0191 (5)	0.0225 (5)	0.0207 (5)	0.0012 (4)	0.0016 (4)	0.0091 (4)
C16	0.0234 (5)	0.0373 (6)	0.0267 (6)	0.0055 (5)	0.0040 (4)	0.0214 (5)
C17	0.0259 (6)	0.0401 (7)	0.0298 (6)	0.0092 (5)	0.0035 (5)	0.0212 (5)
C18	0.0198 (5)	0.0288 (5)	0.0206 (5)	0.0057 (4)	0.0054 (4)	0.0133 (4)
C19	0.0215 (5)	0.0256 (5)	0.0242 (5)	0.0053 (4)	0.0071 (4)	0.0132 (4)
C20	0.0207 (5)	0.0282 (5)	0.0217 (5)	0.0046 (4)	0.0046 (4)	0.0097 (4)
C21	0.0205 (5)	0.0330 (6)	0.0228 (5)	0.0085 (4)	0.0061 (4)	0.0141 (4)
C22	0.0266 (6)	0.0283 (6)	0.0274 (6)	0.0079 (4)	0.0069 (4)	0.0164 (5)
C23	0.0246 (5)	0.0253 (5)	0.0245 (5)	0.0042 (4)	0.0038 (4)	0.0109 (4)
C24	0.0283 (6)	0.0398 (7)	0.0248 (6)	0.0093 (5)	0.0040 (5)	0.0172 (5)
C25	0.0212 (5)	0.0237 (5)	0.0242 (5)	0.0038 (4)	0.0031 (4)	0.0127 (4)
C26	0.0276 (6)	0.0261 (5)	0.0245 (5)	0.0048 (4)	0.0069 (4)	0.0097 (4)
C27	0.0312 (6)	0.0238 (5)	0.0270 (6)	0.0035 (5)	0.0012 (5)	0.0071 (4)
C28	0.0234 (5)	0.0244 (5)	0.0355 (6)	0.0035 (4)	0.0018 (5)	0.0142 (5)
C29	0.0239 (5)	0.0307 (6)	0.0324 (6)	0.0080 (5)	0.0082 (5)	0.0182 (5)
C30	0.0251 (5)	0.0282 (5)	0.0230 (5)	0.0071 (4)	0.0045 (4)	0.0121 (4)
C31	0.0258 (6)	0.0355 (7)	0.0477 (8)	−0.0015 (5)	0.0004 (6)	0.0142 (6)

### Geometric parameters (Å, º)

**Table:**

S1—C8	1.7344 (11)	C14—H14A	0.9800
S1—C11	1.7448 (11)	C14—H14B	0.9800
O1—C7	1.2315 (13)	C14—H14C	0.9800
O2—C12	1.2221 (13)	C16—H16A	0.9900
O3—C12	1.3362 (13)	C16—H16B	0.9900
O3—C13	1.4548 (12)	C16—C17	1.5093 (17)
O4—C15	1.2250 (14)	C17—H17A	0.9800
O5—C15	1.3416 (13)	C17—H17B	0.9800
O5—C16	1.4541 (12)	C17—H17C	0.9800
N1—C1	1.4054 (13)	C18—C19	1.3904 (16)
N1—C18	1.4309 (13)	C18—C23	1.3970 (15)
N1—C25	1.4254 (14)	C19—H19	0.9500
N2—H2	0.850 (17)	C19—C20	1.3977 (15)
N2—C7	1.3644 (13)	C20—H20	0.9500
N2—C8	1.3829 (13)	C20—C21	1.3970 (16)
N3—H3A	0.883 (17)	C21—C22	1.3955 (17)
N3—H3B	0.810 (17)	C21—C24	1.5114 (15)
N3—C11	1.3529 (14)	C22—H22	0.9500
C1—C2	1.4033 (15)	C22—C23	1.3881 (16)
C1—C6	1.4029 (15)	C23—H23	0.9500
C2—H2A	0.9500	C24—H24A	0.9800
C2—C3	1.3799 (15)	C24—H24B	0.9800
C3—H3	0.9500	C24—H24C	0.9800
C3—C4	1.3970 (15)	C25—C26	1.3951 (16)
C4—C5	1.3978 (15)	C25—C30	1.3959 (16)
C4—C7	1.4843 (14)	C26—H26	0.9500
C5—H5	0.9500	C26—C27	1.3921 (17)
C5—C6	1.3885 (15)	C27—H27	0.9500
C6—H6	0.9500	C27—C28	1.3923 (18)
C8—C9	1.3767 (14)	C28—C29	1.3920 (18)
C9—C10	1.4527 (14)	C28—C31	1.5110 (17)
C9—C12	1.4741 (14)	C29—H29	0.9500
C10—C11	1.3902 (15)	C29—C30	1.3854 (16)
C10—C15	1.4647 (14)	C30—H30	0.9500
C13—H13A	0.9900	C31—H31A	0.9800
C13—H13B	0.9900	C31—H31B	0.9800
C13—C14	1.5025 (16)	C31—H31C	0.9800
			
C8—S1—C11	90.70 (5)	O5—C15—C10	114.12 (9)
C12—O3—C13	115.11 (8)	O5—C16—H16A	110.2
C15—O5—C16	115.43 (8)	O5—C16—H16B	110.2
C1—N1—C18	119.80 (9)	O5—C16—C17	107.42 (9)
C1—N1—C25	122.19 (9)	H16A—C16—H16B	108.5
C25—N1—C18	117.44 (9)	C17—C16—H16A	110.2
C7—N2—H2	121.9 (11)	C17—C16—H16B	110.2
C7—N2—C8	125.56 (10)	C16—C17—H17A	109.5
C8—N2—H2	112.5 (11)	C16—C17—H17B	109.5
H3A—N3—H3B	120.2 (15)	C16—C17—H17C	109.5
C11—N3—H3A	119.3 (10)	H17A—C17—H17B	109.5
C11—N3—H3B	114.4 (11)	H17A—C17—H17C	109.5
C2—C1—N1	120.25 (10)	H17B—C17—H17C	109.5
C6—C1—N1	121.47 (10)	C19—C18—N1	120.52 (10)
C6—C1—C2	118.28 (10)	C19—C18—C23	119.47 (10)
C1—C2—H2A	119.6	C23—C18—N1	120.01 (10)
C3—C2—C1	120.74 (10)	C18—C19—H19	120.0
C3—C2—H2A	119.6	C18—C19—C20	120.00 (10)
C2—C3—H3	119.4	C20—C19—H19	120.0
C2—C3—C4	121.26 (10)	C19—C20—H20	119.5
C4—C3—H3	119.4	C21—C20—C19	121.05 (10)
C3—C4—C5	118.13 (10)	C21—C20—H20	119.5
C3—C4—C7	117.52 (10)	C20—C21—C24	121.84 (11)
C5—C4—C7	124.35 (10)	C22—C21—C20	118.02 (10)
C4—C5—H5	119.5	C22—C21—C24	120.13 (10)
C6—C5—C4	121.08 (10)	C21—C22—H22	119.3
C6—C5—H5	119.5	C23—C22—C21	121.46 (10)
C1—C6—H6	119.8	C23—C22—H22	119.3
C5—C6—C1	120.50 (10)	C18—C23—H23	120.0
C5—C6—H6	119.8	C22—C23—C18	119.95 (11)
O1—C7—N2	121.07 (10)	C22—C23—H23	120.0
O1—C7—C4	123.05 (10)	C21—C24—H24A	109.5
N2—C7—C4	115.86 (9)	C21—C24—H24B	109.5
N2—C8—S1	121.56 (8)	C21—C24—H24C	109.5
C9—C8—S1	113.34 (8)	H24A—C24—H24B	109.5
C9—C8—N2	125.00 (10)	H24A—C24—H24C	109.5
C8—C9—C10	111.77 (9)	H24B—C24—H24C	109.5
C8—C9—C12	118.10 (9)	C26—C25—N1	122.05 (10)
C10—C9—C12	129.18 (9)	C26—C25—C30	118.80 (10)
C9—C10—C15	129.14 (9)	C30—C25—N1	119.09 (10)
C11—C10—C9	111.57 (9)	C25—C26—H26	120.0
C11—C10—C15	118.22 (9)	C27—C26—C25	120.10 (11)
N3—C11—S1	118.63 (8)	C27—C26—H26	120.0
N3—C11—C10	128.82 (10)	C26—C27—H27	119.3
C10—C11—S1	112.55 (8)	C26—C27—C28	121.43 (11)
O2—C12—O3	122.47 (10)	C28—C27—H27	119.3
O2—C12—C9	123.55 (10)	C27—C28—C31	121.27 (12)
O3—C12—C9	113.85 (9)	C29—C28—C27	117.78 (11)
O3—C13—H13A	110.2	C29—C28—C31	120.94 (12)
O3—C13—H13B	110.2	C28—C29—H29	119.2
O3—C13—C14	107.46 (9)	C30—C29—C28	121.52 (11)
H13A—C13—H13B	108.5	C30—C29—H29	119.2
C14—C13—H13A	110.2	C25—C30—H30	119.8
C14—C13—H13B	110.2	C29—C30—C25	120.31 (11)
C13—C14—H14A	109.5	C29—C30—H30	119.8
C13—C14—H14B	109.5	C28—C31—H31A	109.5
C13—C14—H14C	109.5	C28—C31—H31B	109.5
H14A—C14—H14B	109.5	C28—C31—H31C	109.5
H14A—C14—H14C	109.5	H31A—C31—H31B	109.5
H14B—C14—H14C	109.5	H31A—C31—H31C	109.5
O4—C15—O5	122.48 (10)	H31B—C31—H31C	109.5
O4—C15—C10	123.32 (10)		
			
S1—C8—C9—C10	2.92 (12)	C10—C9—C12—O2	171.40 (11)
S1—C8—C9—C12	−166.93 (8)	C10—C9—C12—O3	−12.61 (16)
N1—C1—C2—C3	−179.59 (10)	C11—S1—C8—N2	−178.08 (9)
N1—C1—C6—C5	179.10 (10)	C11—S1—C8—C9	−1.65 (9)
N1—C18—C19—C20	179.76 (10)	C11—C10—C15—O4	−25.80 (17)
N1—C18—C23—C22	178.36 (10)	C11—C10—C15—O5	151.08 (10)
N1—C25—C26—C27	−178.50 (10)	C12—O3—C13—C14	−171.23 (10)
N1—C25—C30—C29	176.33 (10)	C12—C9—C10—C11	165.45 (11)
N2—C8—C9—C10	179.21 (10)	C12—C9—C10—C15	−26.80 (19)
N2—C8—C9—C12	9.36 (16)	C13—O3—C12—O2	−3.21 (15)
C1—N1—C18—C19	−52.46 (15)	C13—O3—C12—C9	−179.25 (9)
C1—N1—C18—C23	128.71 (12)	C15—O5—C16—C17	170.77 (10)
C1—N1—C25—C26	−33.06 (16)	C15—C10—C11—S1	−167.48 (8)
C1—N1—C25—C30	149.85 (11)	C15—C10—C11—N3	11.52 (18)
C1—C2—C3—C4	0.17 (17)	C16—O5—C15—O4	−2.93 (15)
C2—C1—C6—C5	−1.21 (17)	C16—O5—C15—C10	−179.84 (9)
C2—C3—C4—C5	−0.57 (17)	C18—N1—C1—C2	−22.84 (16)
C2—C3—C4—C7	178.97 (10)	C18—N1—C1—C6	156.86 (11)
C3—C4—C5—C6	0.07 (17)	C18—N1—C25—C26	138.27 (11)
C3—C4—C7—O1	11.30 (17)	C18—N1—C25—C30	−38.82 (15)
C3—C4—C7—N2	−167.26 (10)	C18—C19—C20—C21	2.63 (17)
C4—C5—C6—C1	0.83 (17)	C19—C18—C23—C22	−0.49 (17)
C5—C4—C7—O1	−169.20 (11)	C19—C20—C21—C22	−1.89 (17)
C5—C4—C7—N2	12.25 (16)	C19—C20—C21—C24	176.92 (10)
C6—C1—C2—C3	0.71 (17)	C20—C21—C22—C23	−0.03 (17)
C7—N2—C8—S1	−1.59 (16)	C21—C22—C23—C18	1.21 (18)
C7—N2—C8—C9	−177.59 (11)	C23—C18—C19—C20	−1.40 (16)
C7—C4—C5—C6	−179.43 (10)	C24—C21—C22—C23	−178.86 (11)
C8—S1—C11—N3	−179.24 (9)	C25—N1—C1—C2	148.29 (11)
C8—S1—C11—C10	−0.12 (9)	C25—N1—C1—C6	−32.02 (16)
C8—N2—C7—O1	1.16 (18)	C25—N1—C18—C19	135.99 (11)
C8—N2—C7—C4	179.75 (10)	C25—N1—C18—C23	−42.84 (15)
C8—C9—C10—C11	−2.98 (14)	C25—C26—C27—C28	2.25 (18)
C8—C9—C10—C15	164.77 (11)	C26—C25—C30—C29	−0.85 (17)
C8—C9—C12—O2	−20.79 (16)	C26—C27—C28—C29	−0.79 (18)
C8—C9—C12—O3	155.21 (10)	C26—C27—C28—C31	−179.45 (11)
C9—C10—C11—S1	1.75 (12)	C27—C28—C29—C30	−1.51 (17)
C9—C10—C11—N3	−179.24 (11)	C28—C29—C30—C25	2.35 (18)
C9—C10—C15—O4	167.14 (11)	C30—C25—C26—C27	−1.40 (17)
C9—C10—C15—O5	−15.98 (16)	C31—C28—C29—C30	177.16 (11)

### Hydrogen-bond geometry (Å, º)

**Table:**

*D*—H···*A*	*D*—H	H···*A*	*D*···*A*	*D*—H···*A*
N2—H2···O2	0.850 (17)	1.958 (17)	2.6501 (12)	137.8 (15)
N3—H3*A*···O1^i^	0.883 (17)	2.154 (17)	3.0316 (13)	172.5 (14)
N3—H3*B*···O4	0.810 (17)	2.156 (16)	2.7656 (13)	132.2 (14)

Symmetry code: (i) −*x*−1, −*y*, −*z*+1.
